# Homomeric and Heteromeric Aβ Species Exist in Human Brain and CSF Regardless of Alzheimer’s Disease Status and Risk Genotype

**DOI:** 10.3389/fnmol.2019.00176

**Published:** 2019-07-31

**Authors:** Erica Lana, Anna Gellerbring, Sabrina Jung, Agneta Nordberg, Christina Unger Lithner, Taher Darreh-Shori

**Affiliations:** ^1^Department of Neurobiology, Care Sciences and Society, Division of Clinical Geriatrics, Center for Alzheimer Research, Karolinska Institutet (KI), Stockholm, Sweden; ^2^Theme Aging, The Aging Brain, Karolinska University Hospital, Stockholm, Sweden

**Keywords:** Alzheimer’s disease, homomeric and heteromeric amyloid-β, apolipoprotein E, cholinesterases, cholinergic signaling

## Abstract

**Background**: A fundamental question in Alzheimer’s disease (AD) is whether amyloid-β (Aβ) peptides and their deposition in the brain signify a direct pathological role or they are mere outcome of the disease pathophysiological events affecting neuronal function. It is therefore important to decipher their physiological role in the brain. So far, the overwhelming focus has been on the potential toxicity of Aβ, often studied outside the crucial AD characteristics, i.e.: (i) the slow, decades-long disease progression that precedes clinical symptoms; (ii) the link to apolipoprotein-E ε4 allele as major risk factor; (iii) the selective early degeneration of cholinergic neurons. Previous studies, *in vitro* and cerebrospinal fluid (CSF) only, indicated one possible native function of Aβ peptides is the allosteric modulation of acetylcholine homeostasis, *via* molecular interactions between Aβ, apolipoprotein-E, and the acetylcholine-degrading enzymes, cholinesterases, resulting in the formation of acetylcholine-hydrolyzing complexes (BAβACs).

**Methods**: Here, by combining sucrose-density gradient fractionation of post-mortem brains and in-house developed sensitive ELISA assays on the obtained fractions, we investigated the presence, levels and molecular interactions between Aβ, apolipoprotein-E and cholinesterases for the first time in brain tissues. We examined three distinct brain regions of Alzheimer and non-demented subjects, plus a large number of Alzheimer CSF samples.

**Results**: We report that both monomeric and oligomeric (homomeric and heteromeric) forms of Aβ peptides are present in the brain of Alzheimer and non-demented individuals. Heteromeric Aβ was found in stable complexes with apolipoprotein-E and/or cholinesterases, irrespective of *APOE* genotype or disease status, arguing in favor of a physiological dynamic formation and function for these complexes in the brain. The patterns and molecular sizes of the detected soluble Aβ forms were closely matched between CSF and brain samples. This evinces that the detected Aβ-apolipoprotein-E complexes and BAβACs in CSF most likely originate from the interstitial fluids of the brain.

**Conclusions**: In conclusion, both light homomeric Aβ oligomers and heteromeric Aβ-ApoE and BAβACs are present and readily detectable in the brain, regardless of disease status and *APOE4* genotype. Deeper knowledge of the physiological function of Aβ is crucial for better understanding the early pathological events that decades later lead to manifestation of AD.

## Background

Alzheimer’s disease (AD) is the most common form of dementia in the elderly population. One of the major pathological hallmarks of the AD brain is the presence of plaques composed of amyloid-beta (Aβ) fibrils. The Aβ peptides and several other fragments are generated through a highly sophisticated and complex enzymatic cleavage of amyloid precursor protein (APP), which occurs up-to-date at four different sites (α, β, η and γ; Willem et al., 2015). The complexity of the APP processing increases even more if one includes the mechanisms involved in the cellular trafficking of APP (O’Brien and Wong, 2011) to and from the cell membranes, the action-potential synchronized synaptic release of Aβ peptides (Cirrito et al., 2005), and the diurnal cycle for Aβ levels in the interstitial fluids in the brain (Kang et al., 2009).

A fundamental question is hence to decipher the physiological function of these peptides in the brain. This, in turn, should allow us to decode how and when the Aβ peptides and/or their effector units turn pathological with regards to the basic neuronal functions and the brain microenvironment.

Yet, the overwhelming focus of the research in the field appears to be on the potential toxicity and pathogenicity of the Aβ peptides, and very few studies are done on deciphering their native biological functions (Fogel et al., 2014; Kumar et al., 2016). During the last decades, the Aβ toxicity paradigm has been shifted from focusing on Aβ fibrils and plaques to the soluble Aβ oligomers and protofibers (Klein et al., 2001). In particular, soluble Aβ oligomers are currently regarded as the culprit for synaptic pathologies and memory deficits observed in AD (Klein, 2002; Walsh and Selkoe, 2004).

In addition, the toxicity of Aβ peptides has been most often studied outside the context and the crucial characteristics of AD, such as: (i) the very slow disease progression that requires several decades to cause discernable pathological manifestations; (ii) its link to advanced age and to the ε4 allele of apolipoprotein-E (*APOE*) gene, as major risk factors; and (iii) the selective and early degeneration of the central cholinergic neurons.

Furthermore, the main utilized technique for showing the existence of the Aβ oligomers has been Western-blotting, which is fundamentally unsuitable for such a purpose. Another prevailing presumption is that Aβ oligomers are homomeric, i.e., the complexes consist only of Aβ peptides (Lambert et al., 2001; Klein, 2002; Gong et al., 2003; Georganopoulou et al., 2005; Esparza et al., 2013; Yang et al., 2013; Cohen et al., 2015). Therefore, the absolute majority of studies (on cell cultures and/or animal tissues) have used Aβ oligomers that are prepared from Aβ40 or Aβ42, and few also a mixture of Aβ40/42 peptides. This is despite the results of numerous studies on postmortem brain tissues, reporting that Aβ deposits are most often accompanied by various other proteins. ApoE protein, and the acetylcholine-degrading enzymes, acetylcholinesterase (AChE) and butyrylcholinesterase (BuChE) are the most prominent examples (Geula et al., 1994; Rees et al., 2003). Reports also exist concerning such molecular interactions between Aβ peptides and ApoE, ApoJ and/or AChE and BuChE proteins *in vitro* and in cerebrospinal fluid (CSF) samples (Ghiso et al., 1993; Strittmatter et al., 1993a,b; Diamant et al., 2006; Darreh-Shori et al., 2011a, b; Kumar et al., 2016). Few studies have also reported possible consequences of such heteromeric molecular interactions, for instance that AChE accelerates (Rees et al., 2003), while BuChE delays Aβ fibrillization (Diamant et al., 2006; Podoly et al., 2008). There is also additional *in vivo* evidence from positron emission tomography (PET) imaging studies that support albeit indirectly the heteromeric molecular interactions between ApoE and/or BuChE and Aβ, with an increased Aβ aggregation/deposit as one of the consequences (Saunders et al., 1993; Schmechel et al., 1993; Kay et al., 2003; Ramanan et al., 2014).

It is also important to recognize that the observations in post-mortem brain as well as the PET studies most likely reflect the endpoint outcome of a slow and subtle long-term interaction between these proteins and Aβ peptides rather than a fast phenomenon. Indeed, it is now well-established that when the clinical symptoms become evident the pathological events have been ongoing for several decades. An unexpected observation reported from this laboratory emphasizes this need for a proper understanding of the temporal and the differential sequence of outcomes of the interaction of other macromolecules with Aβ peptides. While there are no doubts that *APOE4* genotype and ApoE ε4 isoprotein are associated with the higher amount of fibrillar forms of Aβ deposits in the AD brain (Saunders et al., 1993; Schmechel et al., 1993; Kay et al., 2003; Ramanan et al., 2014), we have found that ApoE, in particular the ε4 isoprotein, at physiological concentrations is one of the strongest endogenous anti-Aβ fibrillization agents (Kumar et al., 2016). At the highest tested concentration, which corresponded to the CSF ApoE levels found in *APOE4* homozygotes, ApoE protein prevented Aβ fibrillization for at least 65 h, although the tested Aβ concentration was as high as 20 μM (Kumar et al., 2016). Thus, how and when such a strong anti-Aβ fibrillization activity of ApoE protein changes into an Aβ fibrillization-promoting activity is important to be carefully elucidated.

Overall, all these studies, regardless of being conducted on highly purified proteins, extracts from cell cultures and/or CSF samples confirm one phenomenon beyond any doubt: that a direct molecular interaction occurs between Aβ peptides and ApoE and/or the cholinesterases (AChE and BuChE; Ghiso et al., 1993; Saunders et al., 1993; Schmechel et al., 1993; Strittmatter et al., 1993a,b; Wisniewski et al., 1993; Geula et al., 1994; Mesulam and Geula, 1994; Rees et al., 2003; Diamant et al., 2006; Podoly et al., 2008; Darreh-Shori et al., 2011a, b; Ramanan et al., 2014; Kumar et al., 2016).

Although we have examined, both *in vitro* and in CSF, the formation of heteromeric soluble Aβ-ApoE and BuChE/AChE-Aβ-ApoE (termed as BAβACs), no study is yet available on the formation and/or sources of these soluble heteromeric Aβ complexes in the brain. Here, we investigated the presence of these complexes in the brain homogenates (BH) prepared from frontal, parietal and temporal cortices of the brain of a group of AD and control cases, and also compared the findings with those observed in the CSF of AD patients, in particular in relation to the *APOE4* genotype.

The results indicate that both light homomeric Aβ oligomers (<48 kDa) and heteromeric Aβ-ApoE and BAβACs are present and readily detectable in the brain, regardless of the disease status and *APOE4* genotype. In AD, BAβACs were abnormally hyperactive in both CSF and brain and accompanied by higher ApoE levels. Thus, the current report provides new evidence that both homomeric Aβ and heteromeric complexes of Aβ with ApoE and cholinesterases occur naturally in the brain, and emphasizes the need for further deciphering their possible biological function in the human brain, as well as their possible pathological alterations during the course of AD.

## Materials and Methods

### CSF Pooled Samples and Post-mortem Brain Tissue Homogenates

The CSF samples were prepared as previously described (Vijayaraghavan et al., 2013). Briefly, nine pooled CSF samples obtained from 179 patients with the clinical diagnosis of mild-to-moderate AD with defined *APOE* and *BCHE-K* genotypes were prepared by pooling equal volumes of at least three independent samples with identical genotypes. All samples were collected prior to any cholinesterase inhibitor treatment.

The following nine genotype combinations of pooled samples were prepared:

**Table:** 

**A**	(APOE^ε44^/BCHE^WW^);	**B**	(APOE^ε44^/BCHE^KK^);
**C**	(APOE^ε44^/BCHE^WK^);	**D**	(APOE^ε43^/BCHE^WW^);
**E**	(APOE^ε43^/BCHE^KK^);	**F**	(APOE^ε43^/BCHE^WK^);
**G**	(APOE^ε33^/BCHE^WW^);	**H**	(APOE^ε33^/BCHE^KK^);
**I**	(APOE^ε33^/BCHE^WK^).		

Cortical brain tissues were from medial frontal gyrus (MFG), superior temporal gyrus (STG) and superior parietal gyrus (SPG) of AD patients (*n* = 6, mean age 80.5 ± 8.5, Braak 5–6, post-mortem time 4.98 ± 0.45 h) and of age-matched non-demented controls (*n* = 6, mean age 80.2 ± 7.9, Braak 1–2, post-mortem time 7.72 ± 1.6 h), Table 1. These regions are known to have pathological changes in AD (Connor et al., 1993; Nicoll et al., 2003; Neufang et al., 2011).

**Table 1:** Demographic characteristics for the post-mortem brain tissues.

NBB Sample number	Disease status	Brain region	Duration of disease (y)	Braak stage	PMD (h)	*APOE* genotype	*BCHE* genotype
1995–054	Ctrl	MFG, SPG, STG	-	I	9.2	ε3/ε3	K+/−
1996–085	Ctrl	MFG, SPG, STG	-	I	9.0	ε3/ε3	K−/−
2008–027	Ctrl	MFG, SPG, STG	-	I	7.0	ε4/ε3	K−/−
2008–032	Ctrl	MFG, SPG, STG	-	II	8.9	ε4/ε3	K−/−
2008–054	Ctrl	MFG, SPG, STG	-	I	7.0	ε4/ε3	K +/−
2009–005	Ctrl	MFG, SPG, STG	-	I	5.2	ε2/ε3	K−/−
2004–068	AD	MFG, SPG, STG	5	VI	6.5	ε4/ε4	K−/−
2005–040	AD	MFG, SPG, STG	9	VI	5.0	ε4/ε3	K−/−
2006–059	AD	MFG, SPG, STG	11	VI	3.75	ε4/ε3	K+/−
2007–052	AD	MFG, SPG, STG	8	V	4.25	ε4/ε3	K−/−
2007–073	AD	MFG, SPG, STG	12	V	6.1	ε3/ε3	K−/−
2008–004	AD	MFG, SPG, STG	6	VI	4.3	ε4/ε3	K−/−

*Gender was equally distributed between and within the groups. The mean age was 80 years and ranged 71–92 among the controls and 69–91 among the AD group. Ctrl, Non-demented control; AD, Alzheimer’s disease patient; NBB, Netherlands Brain Bank; MFG, medial frontal gyrus; SPG, superior parietal gyrus; STG, superior temporal gyrus; PMD, post-mortem delay; y, years; h, hours*.

The brain tissues were sequentially homogenized in three different buffers in order to isolate soluble (*s-*), ionic (*i-*), and membrane-bound (*m-*) proteins, as described below. The whole procedure was done at 4°C, excepted when otherwise indicated. The tissue was homogenized in a 15× volume of ice-cold *buffer-I* (50 mM Na/K-phosphate buffer, pH 7.4, prepared from 33 mM Na_2_H_2_ PO_4_(2H_2_O), 17 mM KH_2_PO_4_, 2 mM EDTA), centrifuged at 35,000 *g* for 30 min at 4°C, and the supernatant was carefully transferred to a new tube, and was designated as the *soluble* (*s-*) brain protein extract. Then, the tissue pellet was rehomogenized further in 15× volume of high salt containing *buffer-II* (buffer-I + 500 mM NaCl) and centrifuged as before. The supernatant was transferred to another tube and was designated as the *ionic* (*i-*) brain protein extract. The remaining pellet was rehomogenized once more in 15× volume of *buffer-III* (buffer II + 0.6% (v/v) Triton X-100) and centrifuged as before, and the supernatant was transferred to another tube and was designated as the *membrane-bound* (*m-*) brain protein extract.

The total protein concentration was quantified in all the obtained brain extracts by the *DC*^TM^ Protein colorimetric Assay (Bio-Rad) according to the manufacturer instructions. Serial dilutions of BSA solutions, ranging between 8 mg/ml and 0.0625 mg/ml, were prepared in the different extraction buffers and used as standard concentrations for quantification of total proteins in the corresponding brain extracts.

### Genotyping of Brain Tissue Samples

Pieces of frozen brain tissue with an average weight of 20 mg were cut from the SPG brain region of each individual and the DNA was extracted with a QIAamp DNA Mini Kit (51304, Qiagen) according to the manufacturer’s protocol. Genotyping analyses for *APOE* were performed on the extracted DNA using the TaqMan^®^ GTXpress^TM^ Master Mix (4403311, Applied Biosystems) and the TaqMan^®^ genotyping assays C_3084793_20 and C_904973_10, corresponding to *APOE* SNPs rs429358 (*APOE* ε4) and rs7412 (*APOE* ε2) respectively, according to the manufacturer’s instruction, on a StepOne Plus^TM^ thermal cycler (Applied Biosystems). For *BCHE* genotyping, the rs1803274 SNP was analyzed (assay ID: C__27479669_20), which corresponds to the k-variant of *BCHE*.

### Sucrose Gradient Sedimentation Analysis

Sucrose density gradient (SDG) analysis was used to investigate the presence of homomeric and heteromeric forms of Aβ peptides in the CSF and the brain homogenates. The potential interacting proteins that were assessed were BuChE, AChE, and ApoE proteins, which were previously shown to form stable complexes with Aβ peptides (Kumar et al., 2016). The SDG procedure for pooled CSF samples was described in detail before (Kumar et al., 2016). The SDG procedure for the brain homogenates was the same with minor modifications. Briefly, equal volumes of each brain homogenate (i.e., the *soluble*, *ionic* and *membrane-bound* extracts) were pooled together for each brain region (SPG, MFG, or STG) and for each subject (six AD and six controls). In total 36 pooled brain homogenate samples were examined. Each pooled brain homogenate sample (0.3 mL of sample) was then applied on the top of a transparent Ultra-Clear ultracentrifuge tube (UC, Ultra-Clear^TM^ tube, Cat No. 344059, Beckman, Brea, CA, USA), containing 10 mL of sucrose gradient. In comparison, the amount of the applied pooled CSF samples was 0.5 mL. The gradient was prepared in advance by adding first 5 mL of a 5% sucrose solution (wt/vol) in TBS-MgCl_2_ (10 mM Tris-HCl, pH 7.4, 0.9% NaCl and 50 mM MgCl_2_) to the UC tube. Then 5 mL of a 20% sucrose solution were added by inserting the tip of a Pasteur pipette into the bottom of the UC tube and pipetting the sucrose solution into the upper end of the Pasteur pipette. After sample addition, the UC tube was then capped, rolled on a 45 degree angled surface, and brought to vertical position in a tube holder. The tubes were then subjected to ultracentrifugation for 18 h at 4°C at 165,000 *g* (36,600 rpm) in a Beckman Ultracentrifuge (XL100, rotor SW 41 Ti). Approximately 50 fractions were then collected from the bottom of each tube into a 96-well low-protein-binding microtiter plate that was pretreated with TBS solution containing 0.2% Tween-20 to reduce protein loss from the fractions.

Enzymes of known sedimentation coefficient (S)—bovine liver catalase (11.4S; ~250 kDa, Sigma) and calf intestinal alkaline phosphatase (6.1 S; ~140 kDa, Sigma)—were used in the gradients to estimate the sedimentation coefficient (S) for the individual SDG fractions, using the following formula: S_F_ = (Ft – Fn)/[(Ft – F_i_)*S_i_] where S_F_ is the sedimentation coefficient of a specific fraction, Ft is the total number of fractions from each SDG tube, Fn is the number of the specific fraction of interest, S_i_ and F_i_ are the known sedimentation coefficient and the fraction number showing the highest signal for the internal standard protein, respectively.

The estimated S values were then used to estimate the relative molecular weight for the measured proteins and their complexes detected in the fractions (Mw_F_), using the following formula, known as Svedberg equation: Mw_F_ = (Mw_i_ × S^2^_F_)/S^2^_i_ where Mw_i_ and S_i_ are the known Mw and S values for the internal standard protein (for instance 140 kDa and 6.1 S for calf intestinal alkaline phosphatase).

As previously described (Kumar et al., 2016), we did not observe any molecular interaction between these internal standard proteins and the Aβ, AChE, BChE or ApoE proteins present in the fractions.

### Detection and Measurement of Analytes in the SDG Fractions

#### Quantification of the Aβ_40_ and Aβ_42_ Levels

The relative levels of Aβ_40_ and Aβ_42_ in each collected SDG fraction were measured by an in-house developed sandwich enzyme-linked immunosorbent assay (ELISA). First, a 384-well plate (Nunc MaxiSorp) was coated overnight at 4°C with 50 μL/well of rabbit polyclonal anti-Aβ_40_ antibody (AB5074P, Millipore, Burlington, MA, USA) or rabbit polyclonal antiAβ_42_ antibody (AB5078P, Millipore, Burlington, MA, USA). The working solution of the antibodies was prepared at 1:16,000 in coating buffer (100 mM carbonate buffer pH 9.8: 30 mM Na_2_CO_3_, 70 mM NaHCO_3_, 0.01% Thimerosal). After coating, the plate was washed 1 × 5 min with TBS and blocked for 1 h at room temperature (RT) with 75 μL/well of blocking buffer (4% wt/v of BSA in the coating buffer). After 3 × 5 min washings with 100 μL/well of TBS-T buffer (10 mM TBS, containing 0.05% Tween-20 and 0.01% NaN_3_), 50 μL of standards and undiluted samples were applied to the wells, and the plate was incubated overnight at 4°C. As standards, a 2-fold dilution series of a pooled brain homogenate sample (soluble fractions only), ranging from 660 μg/mL total protein to 645 ng/ml (in TBS, containing 0.05% Triton X-100, 5% sucrose, and 0.01% NaN3) was prepared and used to estimate the relative amounts of Aβ in the SDG fractions. The plate was then washed 3 × 5 min as before with TBS-T, and incubated for 3 h at 37°C with 50 μL/well of working solution of a mouse monoclonal anti-Aβ_1–16_ antibody (SIG-39300, 6E10, Covance; at 1:4,000 dilution in TBS-T buffer, containing 3% BSA). After 3 × 5 min washings as before, the plate was incubated for 2 h at RT with 50 μL/well of working solution of a biotin-conjugated goat polyclonal anti-mouse IgG antibody (B6649, Sigma; at 1:10,000 dilution in TBS-T buffer, containing 3% BSA). The plate was then washed as before and incubated for 2 h with 50 μL/well of a solution containing AP-conjugated streptavidin (11093266910, Roche; 1:5,000 in the TBS-T-BSA buffer). Finally, the plate was washed 4 × 5 min with TBS-T, and 1 × 5 min with DEA buffer (1 M diethanolamine buffer, containing 0.5 mM MgCl_2_•6H_2_O, 0.01% NaN_3_, pH 9.8), and then 50 μL of the AP substrate, p-Nitrophenyl-Na2–6H_2_O (1 mg/ml in DEA buffer) were added and the plate was read at 405 nm, and also at 570 nm (as reference wavelength) by a microplate reader (Infinite M1000, Tecan).

#### Quantification of ApoE Protein Levels

The levels of ApoE protein in the SDG fractions and in the unfractionated brain homogenates were assessed by an in-house developed sandwich ELISA as described before (Darreh-Shori et al., 2011b). Briefly, the general steps were as mentioned above for the Aβ ELISA. The capturing antibody was a mouse monoclonal anti-ApoE antibody (MAB A1.4, Sigma; at 1:8,000 dilution in carbonate buffer, pH 9.8). The 16 standard points were 50 μl/well of a 2-fold serial dilution starting with 2 μg/ml of a purified human ApoE protein (ALP70–50UG, Millipore, Burlington, MA, USA; diluted in 10 mM TBS, containing 0.2% Tween-20 and 0.01% NaN3, pH 7.5). The SDG fraction samples or the unfractionated brain homogenate samples were diluted (1:3) in advance in TBS-T^0.2%^ buffer and added to the wells (50 μL/well). The detecting antibody was a goat polyclonal anti-ApoE antibody (GAB947, Millipore, Burlington, MA, USA; diluted at 1:3,000 in TBS-T^0.05%^, containing 0.5% BSA). The secondary antibody was an alkaline phosphatase-conjugated bovine anti-goat antibody (sc-2353, Santa Cruz; used at 1:2,000 dilution in TBS-T^0.05%^, containing 0.5% BSA). The AP substrate p-Nitrophenyl-Na2–6H_2_O (1 mg/ml in DEA buffer) was added (50 μl/well) to generate a colorimetric reaction that was read by measuring the absorbance at 405 nm in a Tecan Infinite M1000 plate reader.

### AChE and BuChE Activity Measurements

Ellman’s colorimetric assay was used to determine AChE and BuChE activities in the brain homogenates and in the pooled CSF samples. Wells containing the sample buffer or just the Aβ and/or ApoE were used as negative controls. For measuring BuChE activity a master mix (MMB) was freshly prepared and used. MMB contained the BuChE substrate, i.e. butyrylthiocholine iodide (BTC, 5.0 mM final concentration, Sigma), the Ellman’s reagent DTNB (0.4 μM final concentration, Sigma) and the selective AChE inhibitor, BW284C51 (0.001 mM final concentration) in sodium potassium phosphate buffer (50 mM, pH 7.4). For AChE activity, the master mix consisted of the substrate, acetylthiocholine iodide (ATC, 0.5 mM final concentration, Sigma) instead of BTC, and the selective BuChE inhibitor, ethopropazine (0.1 mM final concentration) instead of BW284C51.

In addition to activity measurement, the protein levels of these enzymes in each SDG fraction were determined by ELISA as described before (Darreh-Shori et al., 2006).

### Statistical Analyses

Data are expressed as mean values and standard error of the mean (SEM). Student’s *t*-test was used to compare the levels of biomarkers in the AD and control brain extracts.

## Results

### Distinct Molecular Forms of Aβ Are Present in Both AD and Control Brains

Aβ oligomers have been considered as the most toxic forms of Aβ aggregates, we, therefore, investigated their presence in AD and control brains. To avoid misinterpretation and artifacts we used SDG fractionation of the brain homogenates and analysis of Aβ in the fractions by ELISA.

**Figure 1:**
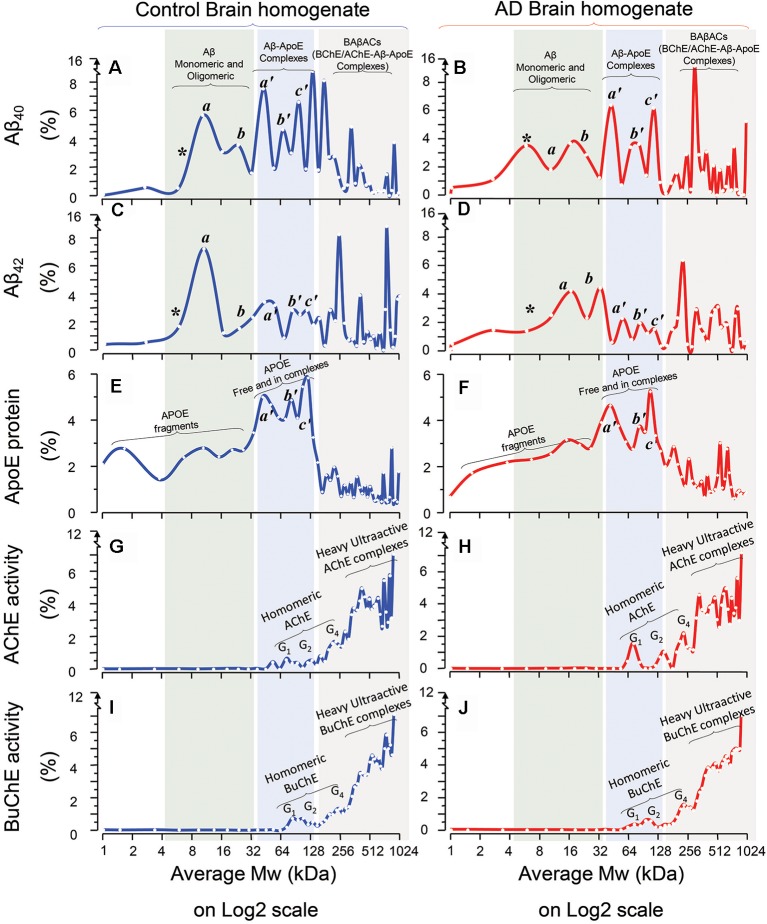
Homomeric and heteromeric forms of Amyloid-beta (Aβ), Apolipoprotein-E (ApoE) and cholinesterases in Alzheimer’s Disease (AD) and non-demented post-mortem brain. Sucrose density gradient (SDG) and ultracentrifugation were used to separate the protein content in the brain homogenates (BH) from a group of AD (*n* = 6) and non-demented controls (*n* = 6), from three different brain regions, medial frontal gyrus (MFG) and/or superior temporal gyrus (STG) and/or superior parietal gyrus (SPG). Following o/n ultracentrifugation, the content of each SDG tube was fractionated into ~50 equal fractions from the bottom of the tubes, and the levels of Aβ_1–40_, Aβ_1–42_, and ApoE proteins, as well as of Acetylcholinesterase (AChE) and Butyrylcholinesterase (BuChE) activities were measured in all fractions, as further described in the “Materials and Methods” section. The y-axis in the SDG spectra indicates the protein level or activity level corresponding to each SDG fraction, represented as % of the total level of that protein in the SDG gradient. The x-axis shows the log2 scale of estimated molecular weight (Mw) corresponding to each fraction content. Each spectrum is composed of aggregated data from the three examined brain regions. **(A–D)** The relative levels of Aβ_1–40_ **(A,B)** and Aβ_1–42_ **(C,D)** peptides in the fractions are plotted vs. the corresponding molecular weights (Mw) of the fractions. The peaks indicated as *, a, and b exhibit Mw values that closely correspond to the molecular weight of Aβ monomers (~4 kDa), dimers (~8 kDa) and tetramers or hexamers (~16–24 kDa) respectively (light green shaded areas). The peaks indicated as a’, b’, and c’ exhibit Mw values that closely correspond to the molecular weight of ApoE monomers (~34 kDa), dimers (~68 kDa) and ApoE complexes (~128 kDa) respectively, which indicates that Aβ peptides were incorporated into stable Aβ-ApoE complexes (the light blue shaded areas). This is confirmed in **(E,F)**, where the levels of ApoE protein are plotted against the Mw values of the fractions. The peaks a’, and b’ exhibit Mw values that closely correspond to the molecular weight of ApoE monomers (~34 kDa) and dimers (~68 kDa) respectively, whereas peak c’ (~128 kDa) indicates heavy ApoE complexes with Aβ (light blue shaded areas). In other words, both ApoE and Aβ SDG spectra indicate that Aβ and ApoE co-sediment and thereby form complexes in the human brain (extracts). **(G–J)** The relative levels of AChE **(G,H)** and BuChE **(I,J)** enzymatic activities in the SDG fractions are plotted against the Mw values of the fractions. The activity peaks indicated as G_1_, G_2_ and G_4_ exhibit Mw values that closely correspond to the expected molecular weight of the monomeric, dimeric and tetrameric globular forms of AChE and BuChE (~55–70, 110, 250 kDa and 75, 150, 280 kDa, respectively). Nonetheless, the most activity-intense peaks show much heavier Mw than the Mw expected for G4 AChE and/or BuChE. A comparison of these heaviest peaks in the SDG spectra among all the panels (gray shaded areas in **A–J**) clearly indicate that these heavy AChE/BuChE molecules co-sediment with certain heavy peaks that contain both Aβ **(A-D)** and ApoE **(E,F)**. Thus, the presence of activity-intense AChE and BuChE peaks at higher Mw fractions (>250 kDa, gray shaded areas) indicates formation of heteromeric complexes of hyperactivated forms of cholinesterases. The presence of high-Mw peaks of Aβ and ApoE in these same areas (graphs **A–F**; gray shaded areas) supports the notion of formation of BAβAC (BuChE/AChE-Aβ-ApoE) complexes in both AD and control brain extracts. Note that there are combinations of technical and mathematical issues that make the use of error bars in the classical meanings inapplicable in an SDG spectrum. This instead is appreciated by considering both the height and width of the peaks in each SDG spectrum, e.g., a comparison between the heights of individual peaks should be avoided unless their width are reasonably similar.

The results are summarized in Figure 1. A comparison between Aβ_40_ SDG diagrams in control (Figure 1A) and AD brains (Figure 1B), revealed the presence of two light peaks corresponding to a Mw range of 4–32 kDa (light green shaded area) in both control and AD brains. Similar twin light-Mw peaks were observed in the Aβ_42_ SDG diagrams (Figures 1C,D). These peaks most likely represent the light homomeric forms of Aβ peptides (monomeric, dimeric, tetrameric and hexa/octameric forms). Noteworthy, the pattern and Mw ranges were very similar between the AD and control brains.

Another noteworthy observation is that in the Aβ light-peak region the control SDG diagrams suggest that the 4–16 kDa Aβ_40_ and Aβ_42_ peaks have higher intensities than the 16–32 kDa Aβ_40_ or Aβ_42_ peaks (compare Figures 1A,C), whilst these peaks have essentially identical intensities in the AD SDG diagrams (Figures 1B,D). In other words, dimeric forms of Aβ_40_ or Aβ_42_ peptides dominate in the control brain, whereas in the AD brain SDG diagrams the peaks corresponding to the dimeric and tetrameric forms of Aβ peptides have similar peak intensities (Figures 1A,C vs. Figures 1B,D).

### Aβ Peptides Interact With ApoE Protein

In addition, the Aβ SDG diagrams showed the presence of a triple peak with moderate Mw, ranging between 32 kDa and 128 kDa (Figure 1; light blue shaded area). Using recombinant Aβ peptides, we have previously shown that Aβ interacts with ApoE protein, producing stable soluble Aβ-ApoE complexes (Kumar et al., 2016). We, therefore, investigated whether similar complexes are formed in the brain. We detected and quantified ApoE protein in the SDG fractions using ELISA. The ApoE SDG diagrams are shown in Figures 1E,F for the control and AD brain homogenates respectively. These diagrams illustrate the presence of a triple peak at Mw range of 32–128 kDa (light blue shaded area), containing high levels of ApoE protein. Peaks corresponding to free ApoE (monomers and dimers) molecules are expected to have Mw of 34–68 kDa. Peaks with larger Mw may hence indicate that some of the ApoE proteins are in complex with other proteins. A comparison between Aβ diagrams (Figures 1A–D) with the ApoE diagrams (Figures 1E,F; light blue shaded area) reveals that Aβ and ApoE proteins co-sedimented as the triple peaks (a’, b’ and c’) in the Mw range of 32–128 kDa. In other words, the SDG fractions corresponding to these Mw contained both Aβ and ApoE, in identical pattern to the Aβ-ApoE complexes previously reported using SDG *in vitro* on recombinant/purified proteins (Kumar et al., 2016). Thus, we conclude that Aβ-ApoE complexes are formed and readily present in the brain. In addition, no apparent differences between the control and AD brain homogenates were found with regard to the presence of these triple peaks (compare Figures 1A,C,E with Figures 1B,D,F).

Interestingly, we also compared Aβ_40_-ApoE with Aβ_42_-ApoE complexes within the control and the AD Aβ SDG diagrams, looking at Aβ peaks in the ApoE-molecular weight areas of the graphs (Figures 1A,B vs. Figures 1C,D, light blue-shaded area). This comparison indicated that a higher proportion of Aβ_40_ peptides were in complex with ApoE than Aβ_42_ peptides in both AD and control brains.

### BAβACs Are Formed and Present in the Brain

Our previous report on SDG analysis on both recombinant proteins and CSF samples has also indicated that BuChE and/or AChE interact with Aβ, particularly in the presence of high ApoE protein, and form BuChE/AChE-Aβ-ApoE complexes (BAβACs). However, experiments using a selective number of recombinant proteins may not be representative of the more complex biomolecular matrix, naturally present in the brain tissues and parenchymal fluids. We hence determined the SDG pattern of AChE (Figures 1G,H) and BuChE (Figures 1I,J) in the brain homogenates from both AD and controls, by measuring the activities of AChE and BuChE in the SDG fractions.

These two cholinergic enzymes are produced as globular (G_1_) subunit, which then assembles from two or four globular subunits to build G_2_ and G_4_ molecular forms of the enzymes. The G_1_ AChE has a molecular weight ranging between 55 kDa and 70 kDa, while G_1_ BuChE is about 75 kDa. Thus, their G_2_ and G_4_ forms are expected to have a Mw of about 110–150 kDa and 250–280 kDa, respectively. In the corresponding SDG diagrams, we indeed observed activity peaks in fractions that exhibited such Mw (Figures 1G–J). However, we also found multiple peaks with molecular weights over 400 kDa to ~1,000 kDa. This strongly suggests that these latter peaks were resulting from complex formation between G_2_ or G_4_ AChE/BuChE and other proteins. A comparison of all the SDG diagrams, specifically within these high-Mw regions (Figure 1, light gray shaded area) shows that ApoE and in particular Aβ also have multiple peaks in this same region, supporting the formation and presence of BAβACs in human brain, in similar pattern as it had been observed following similar analyses performed on recombinant/purified human proteins (Kumar et al., 2016).

### BAβACs Show High ACh-Hydrolyzing Activities

One of the main characteristics of BAβACs is that the incorporated BuChE and AChE enzymes gain a much higher intrinsic ACh-hydrolyzing activity (Kumar et al., 2016). The AChE and BuChE SDG diagrams presented in Figures 1G–J are based on quantitative enzyme activity assessments. Thus, a comparison between the peak intensities of the activity of the homomeric forms of AChE or BuChE (G_1_ to G_4_) and the intensities of the multiple overlapping high-Mw peaks corresponding to BAβACs clearly indicates that the ACh-hydrolyzing activities of the two enzymes are much higher in BAβACs than in the homomeric molecular forms of these enzymes in the brain. This observation also supports the notion of allosteric modulation of ChE activities by Aβ peptides through the formation of BAβACs (Kumar et al., 2016).

The BAβACs hypothesis ascribes the allosteric modulation of ACh-hydrolyzing activity of AChE and/or BuChE as one of the native biological function of Aβ peptides in the brain. This hypothesis thereby predicts that BAβACs should also be present in the brain that is lacking AD pathology. A comparison between the AD and control SDG diagrams indicate that BAβACs are formed and detectable in both AD and control brains, supporting the above hypothesis.

### Differential Presence of ApoE in BAβACs in AD vs. Control Brain

Previous observations suggest that high ApoE protein might be one of the driving factors for abnormal stability and accumulation of BAβACs (Darreh-Shori et al., 2006, 2011b; Vijayaraghavan et al., 2014; Kumar et al., 2016). A comparison between the ApoE SDG diagrams of controls vs. ADs in the SDG region that corresponds to the BAβACs reveals ApoE-containing peaks with higher intensities (peak height) in the AD brain extracts than control (Figure 1E vs. Figure 1F, gray shaded area; 128–1024 kDa Mw range).

**Figure 2:**
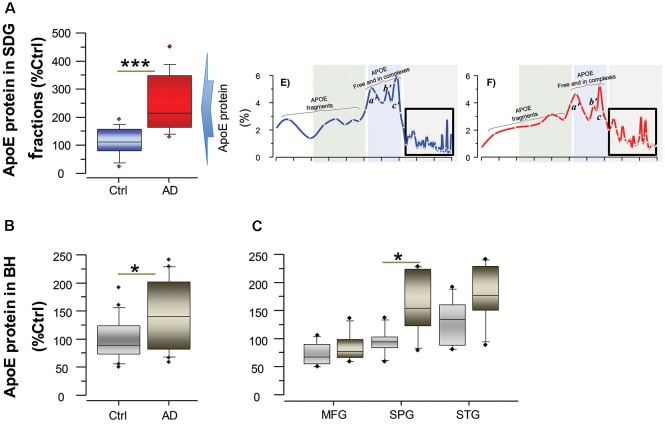
ApoE levels are higher in the brain of AD than controls. ApoE levels were quantified by ELISA both in the SDG fractions **(A)** and in unfractionated brain homogenates **(B)** from the same AD subjects (*n* = 6) and non-demented controls (*n* = 6). Post-mortem brain MFG, STG and SPG regions were used for the measurements. The ApoE levels are presented as percentage of the control brain ApoE. **(A)** Total ApoE protein levels in the SDG fractions (in the three analyzed brain regions), calculated as sum of the ApoE levels in all fractions with Mw >200 kDa, as indicated by the boxed area in the inset SDG spectra from Figures 1E,F; and shown as average of the three analyzed brain regions. **(B,C)** Total ApoE protein levels in unfractionated brain homogenates, shown as average of the three analyzed brain regions **(B)** and in each region separately **(C)**. Student’s *t*-test ***p < 0.001, *p < 0.05.

To further support and complement this finding, we quantified the concentration of ApoE in the AD and control brains both in the SDG fractions, specifically in the SDG region that corresponds to the BAβACs (Figure 2A), and also in the unfractionated brain homogenates from the same individuals (Figures 2B,C). The results showed that ApoE protein levels were indeed significantly higher in the AD than control brains (all *p*-values < 0.05).

### BAβACs May be Dysfunctional in the AD Compared to Control Brain

**Figure 3:**
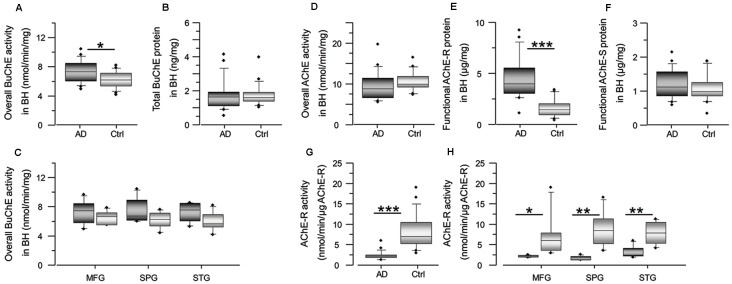
Cholinesterase activity is altered in AD brain, with increase of BuChE activity and decrease of AChE-R activity. Panel **(A)** shows an increase in the overall BuChE activity in the AD brain compared to the control brain homogenates, normalized to the total protein in the brain extracts. The activity was measured by a modified colorimetric Ellman’s assay. Panel **(B)** shows the corresponding total BuChE protein levels in the brain homogenates quantified by sandwich ELISA. Panel **(C)** shows the overall BuChE activity in the AD and the control MFG, STG and SPG brain regions separately. Panel **(D)** shows that the overall AChE activity is similar in the AD brain compared to the control brain homogenates. The activity was measured by a modified colorimetric Ellman’s assay. Panels **(E,F)** show the corresponding protein levels of the functionally intact read-through AChE-R and synaptic AChE-S splice variants of the enzyme in the brain homogenates. These were assayed by a functional semi-sandwich ELISA assay, which utilizes the intrinsic activity of the pre-captured enzymes variants for quantification of their relative amounts in the brain homogenates. It is important to note that these analyses reveal accumulation of inactivated AChE-R variant in the AD brain. This is most simply appreciated from the integrated data of AChE-R activity and protein levels shown in **(G,H)**. The rationale is built on the main methodological difference for data acquisition for graph **(D)** vs. the graph **(E** or **F)**. For graph **(D)**, all the components of the brain extract are present in the well when the activity of the enzyme is measured, while the graphs **(E,F)** are based on activity of the enzymes after capturing the enzyme in the wells, pre-coated with antibody against AChE-R (or AChE-S) variant protein. In other words, in this latter assay all other components present in the brain extracts are washed away. Thus, the higher AChE-R in the AD brain extract in the graph **(E)** means that something in the brain extract had reversibly inactivated the enzyme, and its removal resulted in the re-activation of the enzyme. AD, Alzheimer’s disease (*n* = 6); Ctrl, non-demented controls (*n* = 6); BuChE, butyrylcholinesterase; AChE, acetylcholinesterase; AChE-R, “Read-through” AChE splice variant protein; AChE-S, “Synaptic” AChE splice variant protein. Student’s *t*-test ***p < 0.001, **p < 0.01, *p < 0.05.

One of the characteristics of BAβACs is that high ApoE protein may alter their disintegration leading to gradual accumulation of enzymatically dormant BAβACs, which become readily hyperactivated by excess of Aβ peptide. Considering that Aβ deposits are one of the key features of AD brain, the levels of Aβ peptides are expected to be higher in the AD brain extracts, and consequently BuChE and/or AChE activity might be affected. We, therefore, measured the overall BuChE and AChE activities and protein levels in the control and AD brain homogenates (Figure 3). The results showed that BuChE activity was significantly higher in the AD than the control total brain homogenates (average of the three analyzed brain regions; Figure 3A) and numerically higher in each distinct brain region (Figure 3C). In contrast, the total BuChE protein levels were essentially identical in the AD and control brain homogenates (Figure 3B). These findings are consistent with previous reports in AD CSF (Darreh-Shori et al., 2006, 2011a) and indicate that BuChE exhibits a higher intrinsic catalytic rate per unit of its protein in the AD brain homogenates most likely due to its interaction with Aβ. Thus, high levels of ApoE and Aβ in the AD brain seem to be accompanied by a potentially high degree of abnormal BAβAC functioning.

In contrast to Aβ effect on BuChE, prolonged interaction of Aβ peptides with AChE is shown to lead to reduction in AChE activity and accumulation of Aβ-inhibited AChE protein (Inestrosa et al., 1996a,b, 2008; Darreh-Shori et al., 2014; Kumar et al., 2016), in particular the read-through AChE-R protein variant (Darreh-Shori et al., 2014). Therefore, we also analyzed both the overall AChE activity and the proportion of its functional R and S splice variant proteins in the AD and control brain homogenates (Figures 3D–F). While the overall AChE activity and the protein level of AChE-S variant did not differ between the groups (Figures 3D,F), the AChE-R protein was significantly higher in the AD compared to control brain (Figure 3E). The normalization of the overall AChE activity per unit of AChE-R protein (expressed as nmol/min/μg of AChE-R protein) indicated reduced AChE-R activity in the AD brain extracts (Figures 3G,H). Thus, these results confirmed that AChE-R protein was inactivated in the AD compared to control brain homogenates.

### SDG Spectra Patterns of Homomeric and Heteromeric Aβ Oligomers Are Similar in the Brain and CSF

As noted, ApoE protein levels may play a role in the normal biofunction and/or biodynamic interaction of the interacting partners in BAβACs. In addition, there are indications that ApoE isoproteins may differentially affect the formation of ApoE-Aβ complexes, and thereby the formation of BAβACs (Kumar et al., 2016).

**Figure 4:**
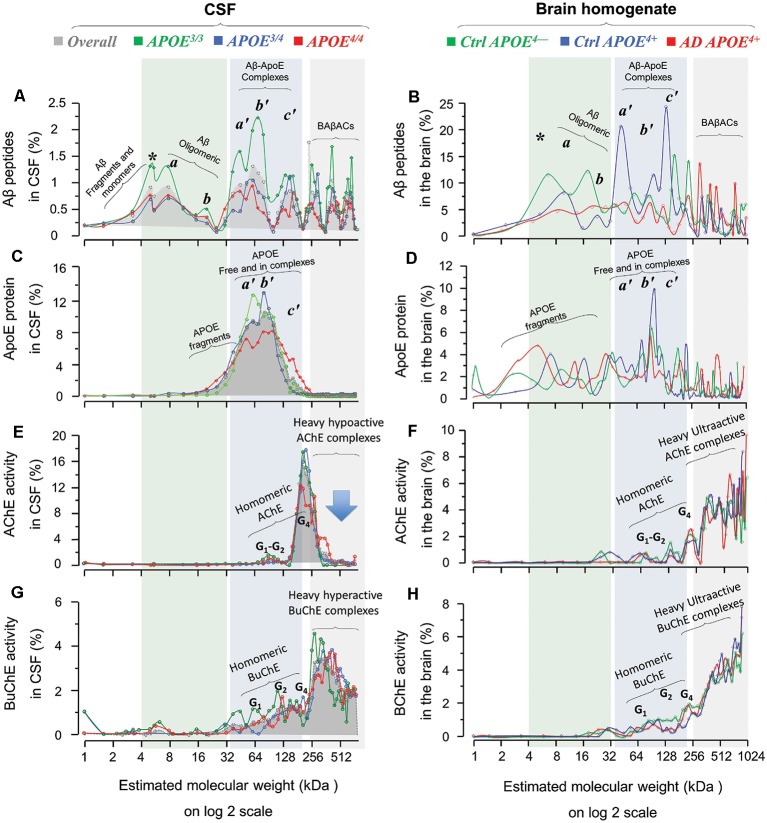
Homomeric and heteromeric forms of Aβ, ApoE, cholinesterases and their complexes in post-mortem brain and cerebrospinal fluid (CSF) in relation to *APOE* genotype. SDG and ultracentrifugation were used to separate the protein content of nine distinct pooled CSF samples, prepared from ~170 AD subjects with known *APOE* genotype (*3/3*, green diagrams; *3/4*, blue diagrams; or *4/4*, red diagrams; in the spectra panels **A,C,E** and **G**). Similar analyses were done on the brain homogenates from a group of AD (*n* = 6) and non-demented controls (*n* = 6), from three different brain regions, MFG and/or STG and/or SPG. Following o/n ultracentrifugation, the content of each SDG tube was fractionated into ~50 equal fractions from the bottom of the tubes, and the levels of Aβ_1–40_, Aβ_1–42_, and ApoE proteins, as well as of AChE and BuChE activities were measured in all fractions, as further described in the “Materials and Methods” section. The y-axis in all SDG spectra indicates the % of protein or activity level in each SDG fraction relative to the total level of that protein in the whole SDG gradient. The x-axis shows the log2 transformation of the estimated molecular weight (Mw) for each fraction. The light green shaded area represents low molecular weight fractions, corresponding to Mw of 4–32 kDa, the light blue shaded area corresponds to Mw of 32 to ~200 kDa, and the light gray shaded area corresponds to Mw ranges between 250 kDa and 1024 kDa. The SDG spectra in **(A)** depict various Aβ-containing peaks separated by SDG ultracentrifugation of pooled AD CSF samples, with color-coded indication of the *APOE* genotype. Panel **(B)** shows Aβ-peaks in the AD and control brain extract samples, with color-coded *APOE* genotype and disease status indication. Comparison of SDG spectra in the light-green shaded area of **(A,B)** reveals essentially similar peak pattern corresponding to Aβ monomers and homomeric Aβ complexes that are detected in both CSF and brain. Peaks marked with * represent Aβ monomers, whereas the peaks a and b correspond to Mw of 8 and ~16 kDa, and may hence represent the homomeric dimers and tetramers of Aβ, respectively. Panels **(C,D)** show the corresponding SDG spectra for ApoE-containing peaks in CSF and brain extracts, respectively. The peaks a’, b’and c’ in the light blue shaded area (Mw range of 32–200 kDa) correspond to both free ApoE protein as well as heteromeric Aβ-ApoE complexes. This is readily appreciated from a comparison between **(A,C)** and between **(B,D)** which confirms the co-sedimentation of Aβ and ApoE in the a’, b’ and c’ Aβ-ApoE triple peaks. Nonetheless, these triple peaks in **(C)** or **(D)** are broad (or less distinct), indicating that a portion of the peaks most likely represents fragments and free monomeric or dimeric ApoE proteins, with an expected size of ~30, 34 and 68 kD. The peak c’ however exhibits a Mw >128 kD, which is clearly too heavy to consist only of ApoE. The SDG spectra in **(E,F)** illustrate the peaks of various globular molecular forms (G_1_, G_2_ and G_4_) of AChE separated based on their relative activity in the SDG fractions of the CSF and brain extracts, respectively. These peaks are accompanied by much heavier AChE-activity peaks (>300 kDa) in both the CSF and the brain SDG spectra. The SDG spectra in **(G,H)** show the corresponding findings for the various BuChE molecular forms (G_1_, G_2_ and G_4_) as well as the heavier (~300–1024 kDa) hyperactive peaks. It should be noted that these heavy AChE and BuChE peaks also exhibit co-sedimentation with the heavy Aβ-peaks (as can be seen in **A,B**), providing evidence for formation of BAβACs in the SDG fractions of both the CSF and brain extracts. However, a major difference is detectable in this region for CSF vs. the brain spectra, particularly in case of the heavy AChE-containing peaks: namely they are relatively hypoactive in CSF (denoted by the arrow in **E**) but are ultra-active in the brain SDG spectra. A more thorough mechanistic explanation for this difference is given in the “Discussion” section, but it should be briefly noted here that this is most likely caused by a prolonged exposure/interaction between Aβ and AChE in CSF which leads to inhibition of AChE (but not BuChE).

We hence investigated the influence of *APOE* genotype and resulting isoproteins on the molecular interactions between Aβ, ApoE and ChEs. However, five out of the six available AD brains were from subjects carrying *APOE4* (four ε3/4 and one ε4/4) genotype. Thereby the brain SDG spectra were sub-analyzed based on three groups of samples: namely *AD APOE4+* (*n* = 5); *Ctrl APOE4+* (*n* = 3) and *Ctrl APOE4−* (*n* = 3). In addition, we performed similar *APOE4* genotype-based analyses on SDG spectra available from a large group of CSF samples from AD patients with known *APOE* genotypes (Figure 4). This way the brain SDG diagrams could be compared along with CSF SDG diagrams.

First, a comparison between the Aβ SDG spectra from the brain homogenates (Figures 1A–D, 4B) and the CSF (Figure 4A) reveals similar peak patterns, regardless of the AD diagnosis or *APOE4* genotype. For instance, the peaks corresponding to the monomers (denoted by *) and the homomeric oligomers of Aβ peptides (denoted as the *a* and *b* peaks) are evident in the CSF and in the AD and control brain homogenates SDG spectra for all three *APOE4* genotype groups (compare the light green shaded areas in Figure 4A with Figure 4B, and/or Figures 1A–D). However, one interesting exception is that the monomeric Aβ peak (* in Figures 1A–D and 4A,B) is more evident in the CSF SDG spectra than in the brain (Figure 4A vs. Figures 1A–D and 4B).

Similarly, the broad triple peaks in the Aβ-ApoE complex area in both Aβ and ApoE SDG diagrams are clearly present in both the brain homogenates and CSF (compare the light blue shaded areas in Figures 1A–F, 4B,D with Figures 4A,C). These findings are important and indicate that the formation of homomeric Aβ oligomers and the heteromeric Aβ-ApoE complexes is a physiologically occurring biodynamic phenomenon as they are formed regardless of the AD diagnosis and the presence or absence of *APOE4* risk allele.

### Levels of Aβ Oligomers, Aβ-ApoE Complexes and BAβACs Are Lower in *APOE4* Carriers in CSF

The similarity of the brain and CSF SDG spectra patterns opens the possibility to use the CSF SDG spectra (which are based on a much larger number of samples) to investigate the influence of *APOE* genotype and resulting isoproteins on the molecular interactions between Aβ, ApoE and ChEs. Indeed, in spite of the pattern similarities among *APOE* genotypes, the intensities of the homomeric and/or heteromeric Aβ peaks exhibited an *APOE*-genotype dependance. The twin peaks (*a* and *b*) corresponding to the homomeric forms of Aβ peptides were relatively more intense in CSF of *APOE*^3/3^ carriers compared to the CSF of carriers of the AD risk allele (*APOE*^3/4^ and *APOE*^4/4^; comparison of the green line SDG spectrum with the blue and red ones in Figure 4A). Similarly, the *APOE*^4/*^ carriers (the blue and red line diagrams) showed lower ApoE and ChE peak intensities, compared to non-carriers (green line) in CSF SDG diagrams (Figures 4C,E,G). This is particularly evident in the CSF of the *APOE*^4/4^ genotype carriers. These observations might be explained by the putative inverse association between CSF Aβ levels and the Aβ deposits in the brain. In other words, lower soluble Aβ, ApoE-Aβ complexes and/or BAβACs are expected to be present in the CSF of *APOE*^4/*^ carriers than in non-carriers (Darreh-Shori et al., 2011a; Kumar et al., 2016), as a consequence of high Aβ deposition in the brain of *APOE*^4/*^ carriers (Darreh-Shori et al., 2011a).

This is also consistent with the finding that ApoE protein is about 130% higher in the AD SDG fractions (with Mw >200 kDa, Figure 2A) and about 50% in the AD brain homogenates (Figures 2B,C) compared to the controls.

## Discussion

In our previous report, we have shown that a diverse range of homomeric and heteromeric forms of Aβ complexes are spontaneously formed (using highly purified proteins) and natively existed in AD CSF samples (Kumar et al., 2016). Here, we used tissue extracts from three different brain regions, namely medial frontal, superior parietal and superior temporal gyri from both AD and control subjects. In agreement with findings *in silico*, *in vitro*, and in AD CSF samples (Kumar et al., 2016), the results indicated that in addition to homomeric (dimeric and tetra-/hexameric) Aβ oligomers, the brain extracts regardless of the disease status also contained several heteromeric Aβ complexes. The findings also provide evidence that the main source of the heteromeric Aβ complexes that were detected in CSF is most likely the brain tissues.

This study thereby confirmed and extended our previous findings by showing for the first time evidence that similar homomeric and heteromeric forms of Aβ complexes natively existed in the brain of patients with AD and non-demented individuals. The overall pattern of the SDG spectra for the types of homomeric and heteromeric Aβ complexes in the brain was also very similar between patients with AD and non-demented individuals. Nonetheless, an overall as well as an *APOE* genotype-specific comparison revealed, besides a high consistency between the overall SDG spectra patterns in AD CSF and the AD and control brain homogenates, certain qualitative differences in the distribution and levels of homomeric and heteromeric Aβ oligomeric forms among the AD APOE4+, the Ctrl APOE4+ and the Ctrl APOE4− groups. These warrant further investigation since such differences may be crucial for understanding the factors that render the homomeric and/or heteromeric Aβ species pathogenic in individuals at risk of AD.

A longstanding question for us concerns the early and selective degeneration of the central cholinergic system in AD, and its link to Aβ peptides, to advanced age and to *APOE4* genotype. Therefore, the current study similarly to the previous ones focused on analyzing the heteromeric nature of Aβ peptides in the SDG fractions with regards to ApoE protein and to two known acetylcholine-degrading enzymes, AChE and BuChE. It is hence noteworthy that the current findings do not exclude the possibility for Aβ forming heteromeric complexes with other interacting partners. Indeed, a large number of functional studies on various preparations of Aβ peptides indicate that Aβ also affects other neuronal systems, and cellular pathways signaling machineries such as N-Methyl-D-aspartate (NMDA) receptors, even though such functional outcomes are most often perceived as justification for toxicity of Aβ peptides (for a relevant discussion see Benilova et al., 2012).

Nevertheless, we found that Aβ oligomers natively existed as soluble homomeric forms, i.e., consisting only of Aβ peptides, as well as soluble heteromeric forms, in which Aβ peptides formed highly stable complexes with ApoE protein and/or the cholinergic enzymes AChE and BuChE. This was true for both CSF and the brain extracts regardless of *APOE4* genotype and/or AD disease status. All the detected Aβ species in the current study “natively” existed/formed since we used as optimized conditions as possible in the preparation of the sucrose gradient solution. One was the omission of detergent in the buffer of sucrose solution to avoid altering the Van der Waals and/or the hydrophobic forces that are crucial in formation of Aβ oligomers as no report exists for the presence of covalent bonding in Aβ oligomers. Similarly, all the detected Aβ complexes were “highly stable” since they apparently resisted disintegration upon exposure to a continuous ultra-centrifugal force for 16–18 h at 4°C. Also, all were “soluble” since none sedimented to the bottom of the SDG tubes.

Altogether, the overall similarity in the diversity, complexity, solubility and stability of the detected monomeric as well as the homomeric and heteromeric forms of Aβ in the AD CSF and the brain extracts of AD and controls, reinforces the idea that they occur natively in the brain regardless of disease status, and may hence represent native functional units required in a healthy brain. It should be however emphasized that these findings do not suggest that Aβ oligomers or simply Aβ peptides are irrelevant but that just being oligomers does not make them pathological or causes AD. The findings imply that our efforts need to be focused on deciphering the native physiological functions of these peptides rather than on defining one as the culprit of AD. A crucial approach is hence to identify the effector units of Aβ peptides and elucidate the factors and conditions that can render their biological action pathological.

In this context, ApoE protein may play a crucial role. Our previous report has shown that ApoE protein facilitates the formation of heteromeric Aβ complexes, most likely by chaperoning the physical interaction between Aβ peptides and the ACh-degrading enzymes BuChE/AChE, resulting in the formation of a functional unit, termed BAβACs (BuChE/AChE–β-amyloid–APOE complexes; Kumar et al., 2016). The main characteristic of the BAβACs is an Aβ-induced allosteric modulation of the activity of the incorporated enzymes, BuChE and AChE (Kumar et al., 2016). Our hypothesis is that BAβACs, as dynamic functional units, are continuously formed and disintegrated in the interstitial fluids of the brain. They are required for the regulation of functional status of cholinoceptive astroglial cells, which most often reside far from the cholinergic synapsis. The regulation of their functions requires, therefore, a dynamic adjustment of an extrasynaptic ACh equilibrium state in the brain (Darreh-Shori et al., 2013; Vijayaraghavan et al., 2013; Malmsten et al., 2014). Thereby neuronal release of Aβ promotes the formation of hyperactive BAβACs, which reduce extrasynaptic ACh levels, resulting in temporal activation of astroglial cells. Reuptake of Aβ peptides, in contrast, promotes the disintegration of the hyperactive BAβACs, allowing the original ACh equilibrium to be reestablished, and thereby forcing astroglial activity back to its original state. Thus, ACh, depending on its concentration, acts either as an activator or a suppressor of activity of non-neuronal cholinoceptive cells *via* its α7 nicotinic receptors (Darreh-Shori et al., 2013; Vijayaraghavan et al., 2013). Nonetheless, additional reports bring in two other major components of the cholinergic machinery into the picture, namely the high affinity choline transporter, hChT (Cuddy et al., 2017) and the ACh biosynthesizing enzyme, ChAT (Dobransky et al., 2003; Kumar et al., 2018). Cuddy et al. (2017) have found that the N-terminal (amino acids 1–16) of Aβ peptides binds to and inhibits hChT (Cuddy et al., 2017), which is responsible for cellular reuptake of choline (Cuddy et al., 2017). Dobransky et al. (2003) have reported that Aβ42 peptides somehow alter the phosphorylation state of ChAT protein, which results in several folds increase in the activity of this ACh-biosynthesizing enzyme (Dobransky et al., 2003). Other reports show that Aβ, in particular Aβ42, peptides are able to allosterically alter the catalytic efficiency of ChAT (Nunes-Tavares et al., 2012; Kumar et al., 2018). Thereby Aβ peptides are able to modulate the activity of ChAT protein both directly and indirectly. Overall, an apparent outcome of the interaction between Aβ and all these cholinergic macromolecules is an alteration in the functional properties of these enzymes and the transporter (Geula et al., 1994; Mesulam and Geula, 1994; Darreh-Shori et al., 2011a, b, 2014; Kumar et al., 2016; Cuddy et al., 2017; Kumar et al., 2018). Intriguingly, Aβ peptides seem to be able to also promote translocation of a certain ChAT variant to the nucleus (Albers et al., 2014), which consequently changes the APP gene expression (Albers et al., 2014; Winick-Ng et al., 2016), indicating that at least the interaction of Aβ with ChAT has bidirectional consequences. Overall, these observations prompted us to update our hypothesis by proposing that at least one of the innate physiological functions of Aβ peptides is modulation of acetylcholine homeostasis in the brain (Figure 6 in Kumar et al., 2018).

Another characteristic of the BAβACs is that they are highly dynamic complexes with no predetermined size, but approximately range between 400 kDa and 1,000 kDa, therefore we have defined them as *Light*, *Heavy* and *Ultra-heavy* BAβACs (Kumar et al., 2016). This characteristic is particularly important for understanding their abnormal function in AD. We have previously hypothesized that abnormally high levels of ApoE protein pathologically alters the normal biodynamics of the interaction between Aβ and BChE/AChE by preventing disintegration of BAβACs. The basis for this hypothesis is that we have found that ApoE protein levels are highest (~150%) in CSF of patients carrying the ε4 allele (ε 4/4 >> ε 4/3> ε 3/*) (Darreh-Shori et al., 2011b), and that a high ApoE protein level abnormally affects the function, stabilization and accumulation of BAβACs (Darreh-Shori et al., 2011a,b, 2013; Kumar et al., 2016). In a mechanistic view, this means that such an abnormal stabilization of BAβACs results in gradual accumulation of these complexes in the CSF and the interstitial fluids of the brain of patients with AD (Darreh-Shori et al., 2006, 2011a,b, 2014; Kumar et al., 2016). This allows BAβACs to increase in their sizes from the normal *Light* forms to the *Ultra-heavy* forms of BAβACs, which sooner or later will become deposited within the Aβ deposits in the AD brain. This, in turn, explains the presence of ApoE, AChE and BuChE in the Aβ deposits (Mesulam and Geula, 1994; Ramanan et al., 2014), in particular in the brain of AD patients carrying *APOE* ε4 allele (Lehmann et al., 1997, 2000). Thus, deposition of ApoE-Aβ complexes and BAβACs within Aβ plaques in the AD brain represents the gradual outcome of such molecular interactions as end-point signs of decades of malfunctioning Aβ-effectors complexes such as BAβACs and the related abnormality in acetylcholine homeostasis in the brain.

A comparison between the pattern of SDG diagrams for both ApoE-Aβ and BAβACs in the CSF and the brain extracts revealed two main differences that are highly relevant in this context. First, the BAβACs in CSF showed reduced activity (or appeared dormant) as the complexes became heavier, whereas in the brain extracts these complexes showed increased catalytic rate as they grew larger (see Kumar et al., 2016 and compare Figure 4E or 4G with Figures 4F and 4H in the current article). This reflects a differential time-dependent effect of Aβ on the activity of AChE compared to BuChE that are incorporated in the BAβACs (Darreh-Shori et al., 2014). During short-term (up to 4 h), interaction of Aβ peptides with AChE and BChE increases the apparent activity of both enzymes as assessed in CSF samples or in solutions of purified enzyme proteins (Darreh-Shori et al., 2014; Kumar et al., 2016). However upon longer interaction time, AChE but not BuChE activity is inhibited by Aβ peptides particularly in presence of high ApoE protein (Darreh-Shori et al., 2014; Kumar et al., 2016). Thus, the initial Aβ-induced increase in BuChE activity remains elevated regardless of the exposure time to Aβ (Darreh-Shori et al., 2014). Given that Aβ and these two enzymes (partly in the form of BAβACs) coexist in CSF, and that the heaviest BAβACs represent the oldest complexes, it is expected that AChE activity is inhibited by Aβ in the oldest complexes, thereby the *Ultra-heavy* forms of BAβACs display a latent (inactivated) phenotype as is depicted in the AChE SDG spectra (Figure 4E), and to a lesser degree in the BuChE SDG spectra (Figure 4G) in AD CSF. In contrast, the pools of AChE, BuChE and ApoE proteins as well as those of Aβ peptides are most likely separated in intact brain tissues/cells but becomes mixed during the homogenization of the brain tissues, allowing Aβ, ApoE and ChEs pools to interact and form BAβACs. Thus, both enzymes, in particular AChE, are expected to be in hyperactive states in the freshly formed BAβACs, as is depicted in the corresponding SDG spectra for the brain extracts (Figures 4F,H; the 256–1024 kDa regions). It should be emphasized here that these specific differences reflect the differences between the nature of CSF and the brain extracts and the disease status because CSF is a naturally occurring extracellular fluid but the used brain extracts are not.

Another informative difference between the SDG pattern of ApoE-Aβ and BAβACs in CSF and in the brain extracts is that much more ApoE protein was found present in the *Heavy* and *Ultra-heavy* BAβACs in the brain homogenate compared with the CSF BAβACs, which seemed to contain very little (if any) ApoE protein (compare the light gray shaded region in the Figure 4D with 4C, in the current article). This observation supports the notion that ApoE acts as a chaperon for interaction between Aβ and ChEs, by suggesting that ApoE will be gradually displaced out as the complexes are maturing/growing in their size. On the other hand, a quantitative comparison of ApoE-containing peaks, in regions corresponding to the BAβACs peaks (boxed in the Figure 2A) in the brain SDG spectra between the control and the AD brain indicated that BAβACs contained more ApoE protein in the AD brain homogenate than in the controls. We also found that the amount of ApoE protein was higher in the total AD brain extracts compared to the control samples, in agreement with CSF findings (Darreh-Shori et al., 2011a,b). These observations are important since they indicate a high ApoE protein resulted in excessive ApoE chaperoning effect on the formation of BAβACs in the AD brain homogenates. In support of this notion, we found a higher amount of hyperactivated AChE-R protein in the AD brain extracts compared to the control brain extracts, thereby indicating a higher degree of BAβACs formation in the AD brain extracts. As might be expected the activity but not the protein level of BuChE was also significantly higher in the AD than the control brain homogenates, indicating a larger number of hyperactivated BuChE were formed in the AD cases when the tissues were homogenized. Overall, while BAβACs as effector units of Aβ represent one possible innate functions of Aβ peptides required for modulation of ACh homeostasis in the brain, a high ApoE protein (mainly as a function of the AD risk allele of *APOE*) represents a possible condition that may render this innate Aβ activity pathogenic.

Noteworthy, high ApoE protein in CSF of AD patients has been found to be associated with dementia and *in vivo* pathological signs of AD (Darreh-Shori et al., 2011a; Vijayaraghavan et al., 2014). These observations have further been confirmed by a study in a large well-characterized population of patients with subjective cognitive decline (SCD) or mild cognitive impairment (MCI). The study further suggests that high CSF ApoE predicts the clinical progression in *APOE* ε4-carriers (van Harten et al., 2017). Thus, our findings provide a mechanistic explanation by suggesting that abnormally high levels of ApoE protein in the brain may mediate, through excessive formation of ApoE-Aβ chaperone units, an abnormal formation, accumulation and stabilization of BAβACs in the interstitial fluids in the brain. This is expected to cause a gradual and detrimental alteration of the delicate dynamic association and dissociation of Aβ with ChEs in forming a functional unit for maintaining a proper ACh signaling, most likely for both cholinergic neurotransmission and extrasynaptic action of Ach (Darreh-Shori et al., 2011a,b; Vijayaraghavan et al., 2014; van Harten et al., 2017). Furthermore, observational studies on the use of cholesterol-lowering drugs indicate that they may be protective against development of AD and/or Aβ pathology (Shepardson et al., 2011). Given that reducing cholesterol and lipid synthesis may also decrease the physiological demand on ApoE protein expression, and thereby at least indirectly reduce the levels of ApoE, our findings suggest that the protective effect of these drugs against AD development may be mediated through a reduced Aβ-ApoE and BAβACs accumulation. Indeed, there is evidence of increased AChE activity in patients with familial hypercholesterolemia and in animal models of hypercholesterolemia (Moreira et al., 2014; Lopes et al., 2015). Altogether, the previous and current findings provide solid scientific premises suggesting that development of effective ApoE lowering drugs is a highly viable preventive strategy against the development of AD. However, it is difficult to predict whether such drugs would be able to show measurable efficacy if they are tested in patients already suffering from AD. The best study populations are patients with SCD or very mild MCI if such drugs have to be tested in patients with clinical symptomatology.

Aβ oligomers are considered the toxic species and the culprit actors in AD (Klein, 2002). However, the definition of Aβ oligomers, their molecular nature, their concentrations and the techniques that most often are employed to access these characteristics need to be critically revised. In the current work and a previous study we showed that the SDG fractionation technique is highly feasible, suitable and superior to achieve the aforementioned information, in particular since the separation occurs without major manipulation of the native conditions of the proteins in the samples. Several studies have reported that the amount of Aβ oligomers in the brain extracts and/or CSF are either in very low (<1 pM and <6.3 pg/mL) ranges or simply under detection level of the conventional quantitative assays (Georganopoulou et al., 2005; Esparza et al., 2013; Yang et al., 2013). In strong contrast, we showed here that an in-house ELISA assay, with regular sensitivity, readily detected a large number of various Aβ species (with apparent Mw corresponding to monomeric, dimeric and tetrameric species, up to as large as 1,000 kD complexes). The majority of studies on Aβ oligomers have used either immunoprecipitation (IP) or Western-blot (WB) techniques on brain homogenates, CSF or cell extracts. These techniques require either pre-clearance and/or mixing the samples with a buffer, which often contains high concentration of a reducing agent and/or high concentration of various detergents, such as Triton X-100 or sodium dodecyl sulfate (SDS), followed by heat denaturation of proteins in the samples (Lesné et al., 2013). The use of such detergents, in particular SDS is highly problematic and may cause artificial aggregation (Barghorn et al., 2005) or disaggregation of the native Aβ complexes that the current study and our previous reports have shown to exist/form *in vitro* and *ex vivo* in CSF and brain extracts (Darreh-Shori et al., 2014; Kumar et al., 2016). Furthermore, the presence of various forms of Aβ oligomers that most often inferred to be the homomeric variants is depicted as more or less distinct bands on the WB membrane, immunostained with anti-Aβ antibodies. The migration pattern of the Aβ bands corresponds to Mw ranging from 8 kDa to ~200 kD. To the best of our knowledge very few (if any) have used double staining of the bands to ascertain that they were really representing only homomeric forms of Aβ complexes. One study by this laboratory, however, used double staining with an anti-Aβ antibody on a set of WB membranes that had been previously used for detection of AChE protein in SDG fractions of CSF samples (Darreh-Shori et al., 2014). The study showed that the anti-Aβ antibody positively stained a heavy band that had been also immunodetected with an anti-AChE antibody. This not only supports our findings but also strongly highlights the lack of consideration about the possibility for the heteromeric nature of Aβ oligomers in the field.

The light homomeric forms of Aβ oligomers have been claimed difficult to detect and isolate in brain extracts, and particularly in CSF (Georganopoulou et al., 2005; Esparza et al., 2013; Yang et al., 2013). This study and another recent one by this laboratory showed that these Aβ oligomers were readily detected following SDG fractionation, regardless of whether the analyzed samples were a mixture of purified proteins (Aβ with ApoE and/or ChEs), human CSF, or brain tissue extracts (Kumar et al., 2016). Another group has successfully used a reasonably similar ultracentrifugation technique on brain extracts (Esparza et al., 2016). Although they also detected Aβ complexes with Mw of about 670 kDa (in the range of BAβACs), their focus has been limited to the detection and isolation of the light homomeric Aβ oligomers of a defined size, and they did not analyze the heteromeric complexes, nor used control brain homogenates for comparison but for testing the stability of the isolated Aβ oligomers (Esparza et al., 2016).

## Conclusion

In summary, the current study provided novel evidence that distinct Aβ_40_ and Aβ_42_ oligomers did natively exist in the brain of both AD and non-demented individuals. This was true for both homomeric and heteromeric forms of Aβ_40_ and Aβ_42_, indicating that they have a physiological role in the healthy brain, in addition to be abnormally involved in pathological processes. This study also provided evidence that the origin of Aβ-ApoE and BAβAC complexes in CSF is most likely from the interstitial fluids in the brain. The readily detectable presence of such a large number of Aβ species implies that the homomeric and heteromeric formation of Aβ oligomers is an innate dynamic process, which is largely independent of the disease status as they formed in the brain extracts of AD patients and non-demented individuals.

## Data Availability

The datasets for this manuscript are not publicly available because the raw data generated and analyzed during this study are available from the corresponding author upon reasonable request. Requests to access the datasets should be directed to taher.darreh-shori@ki.se.

## Ethics Statement

This study was conducted according to the Declaration of Helsinki and subsequent revisions. All studies involving human subjects were approved by the Regional Ethical Review Board in Stockholm or the Research Ethics Committee of the South Huddinge University Hospital. The brain tissues were obtained from Netherlands Brain Bank (NBB), Netherlands Institute for Neuroscience, Amsterdam. The NBB abides by the Dutch law for obtaining and using human tissues for scientific research. All material had been collected from donors from whom a written informed consent for brain autopsy and the use of the material and clinical information for research purposes had been obtained by the NBB.

## Author Contributions

The concept and design of the study was done by TD-S. EL, AG and SJ performed the experiments. EL and TD-S analyzed the data. EL, TD-S and CUL wrote the manuscript. AN provided critical input during the study and during the preparation of the manuscript. All authors read and approved the final manuscript.

## Conflict of Interest Statement

The authors declare that the research was conducted in the absence of any commercial or financial relationships that could be construed as a potential conflict of interest.

## Abbreviations

Aβ: Amyloid-β
ACh: Acetylcholine
AChE: Acetylcholinesterase
AD: Alzheimer’s Disease
APP: Amyloid precursor protein
APOE: Apolipoprotein-E gene
APOE4: ε4 allele of APOE gene
BuChE: Butyrylcholinesterase
BCHE-K: K variant of BCHE gene
BAβACs: BuChE/AChE-Aβ-ApoE complexes
ChEs: Cholinesterases
CSF: Cerebrospinal fluid
ELISA: Enzyme-Linked-Immunosorbent Assay
MCI: Mild cognitive impairment
MFG: Medial frontal gyrus
Mw: Molecular weight
NBB: Netherlands Brain Bank
PET: Positron emission tomography
SCD: Subjective cognitive decline
SDG: Sucrose density gradient
SPG: Superior parietal gyrus
STG: Superior temporal gyrus
WB: Western-blot.
